# Weighted correlation network analysis revealed novel long non-coding RNAs for colorectal cancer

**DOI:** 10.1038/s41598-022-06934-w

**Published:** 2022-02-22

**Authors:** Sepideh Chodary Khameneh, Sara Razi, Sara Shamdani, Georges Uzan, Sina Naserian

**Affiliations:** 1 Department of Molecular and Cellular Sciences, Faculty of Advanced Science and Technology, Tehran Medical Sciences, Isalamic Azad University, Tehran, Iran; 2 Department of Biology, Science and Research Branch, Islamic Azad University, Tehran, Iran; 3 Shahid Beheshti University of Medical Sciences, Tehran, Iran; 4 INSERM UMR-S-MD 1197, Hôpital Paul Brousse, Villejuif, France; 5 Paris-Saclay University, Villejuif, France; 6 CellMedEx, Saint Maur Des Fossés, France; 7 ScreenCell, Sarcelles, France

**Keywords:** Bioinformatics, Gene expression analysis, Medical research, Oncology

## Abstract

Colorectal cancer (CRC) is one of the most prevalent cancers worldwide, which after breast, lung and, prostate cancers, is the fourth prevalent cancer in the United States. Long non-coding RNAs (lncRNAs) have an essential role in the pathogenesis of CRC. Therefore, bioinformatics studies on lncRNAs and their target genes have potential importance as novel biomarkers. In the current study, publicly available microarray gene expression data of colorectal cancer (GSE106582) was analyzed with the Limma, Geoquery, Biobase package. Afterward, identified differentially expressed lncRNAs and their target genes were inserted into Weighted correlation network analysis (WGCNA) to obtain modules and hub genes. A total of nine differentially expressed lncRNAs (LINC01018, ITCH-IT, ITPK1-AS1, FOXP1-IT1, FAM238B, PAXIP1-AS1, ATP2B1-AS1, MIR29B2CHG, and SNHG32) were identified using microarray data analysis. The WGCNA has identified several hub genes for black (LMOD3, CDKN2AIPNL, EXO5, ZNF69, BMS1P5, METTL21A, IL17RD, MIGA1, CEP19, FKBP14), blue (CLCA1, GUCA2A, UGT2B17, DSC2, CA1, AQP8, ITLN1, BEST4, KLF4, IQCF6) and turquoise (PAFAH1B1, LMNB1, CACYBP, GLO1, PUM3, POC1A, ASF1B, SDCCAG3, ASNS, PDCD2L) modules. The findings of the current study will help to improve our understanding of CRC. Moreover, the hub genes that we have identified could be considered as possible prognostic/diagnostic biomarkers. This study led to the determination of nine lncRNAs with no previous association with CRC development.

## Introduction

Colorectal cancer (CRC) is one of the most prevalent digestive system cancers worldwide. After breast, lung, and prostate cancers, it is the fourth prevalent cancer in the United States^1^. Nearly two million new CRC cases and 700,000 deaths from this cancer are reported every year^2^. Its incidence and mortality rate is 25% higher in men than women, and most of the reported CRC cases are in the colon, while fewer cases are in the rectum^3^. Thanks to novel diagnostic tools and therapeutic interventions, the mortality rate has decreased significantly from 1999 to 2015^4^. Although most reported cases of CRC are sporadic (70%), a significant part of the cases occur in patients with familial history of CRC (25%) or hereditary colorectal cancer syndromes (~ 10%)^5^. Therefore, further studies are still required to clarify the involved molecular mechanisms in CRC.

Non-coding RNAs (ncRNAs) are small molecules that are not translated to proteins and participate in various regulatory functions of the cell. They consist of different families including, microRNAs (miRNAs), small nuclear RNAs (snRNAs), PIWI-interacting RNAs (piRNAs), and long non-coding RNAs (lncRNAs)^6^. LncRNAs were recently identified as regulatory molecules with a length of more than 200 nucleotides. They bear some resemblances with mRNAs, including a cap at the 5′ end, having more than one exon, being transcribed by RNA polymerase II (RNA pol II), and being located in the cytoplasm or the nucleus. However, this class has some dissimilarities with mRNAs, including lower expression level, poorer conservation among other species, inability to be transcribed to a protein, and tissue/stage-specific expression^7^. It has been reported that lncRNAs may be up/down-regulated in cancerous cells compared to healthy ones, indicating the possible role of these molecules as an oncogene or a tumor suppressor^8^.

LncRNAs are involved in carcinogenesis and progression of CRC^9–11^. It has been demonstrated that lncRNAs regulate various cellular functions related to CRC pathogenesis, including cell proliferation, apoptosis, migration, invasion, metastasis, differentiation, DNA damage, drug resistance, epithelial-mesenchymal transition (EMT), development, controlling cancer stem cells, and cell cycle^12^. Thanks to high-throughput methods and novel bioinformatics approaches, such as microarray and RNA-seq, many lncRNAs with altered expression in CRC cells have been identified^13^. Forrest et al*.* identified more than 200 differentially expressed lncRNAs by analyzing RNA sequencing data from The Cancer Genome Atlas (TCGA) dataset. Moreover, they concluded these lncRNAs regulate cell cycle genes and increase resistance to apoptosis^14^. Studies have reported that some lncRNAs are significantly overexpressed in the CRC cells and tissues, correlating with metastasis and weak patient prognosis^15^. In addition to CRC cells, lncRNAs with altered expression level has been reported in peripheral blood components such as serum or plasma^16^.

Weighted gene co-expression network analysis (WGCNA) is an in-silico system biology tool to analyze gene expression in a complex network of regulatory genes. This tool based on R programming can identify clusters of highly correlated genes (modules) based on genetic correlations^17^. Therefore, it is helpful for identifing novel diagnostic and prognostic biomarkers for cancer. Zhou et al. reported a number of hub genes and miRNAs which was associated with stages of CRC^18^. In the current study, the WGCNA algorithm was employed to construct a co-expression network of lncRNAs associated with CRC and their target genes. This study would help identify possible new biomarkers for CRC and reach a better understanding of the molecular pathways contributing to this disease.

## Methods

### Data acquisition and processing of lncRNA expression profiles

Microarray gene expression data of colorectal cancer with the series number GSE106582 was obtained from the publicly available Gene Expression Omnibus (GEO) database to identify lncRNA candidates (https://www.ncbi.nlm.nih.gov/geo/query/acc.cgi?acc=gse106582) .GSE106582 was provided by the University clinics Freiburg (Freiburg, Germany), and CRC patients were recruited at the University Hospital of Heidelberg. Total RNA from 77 tumors and 117 mucosa samples were analyzed, including 68 tumor-mucosa pairs using Illumina HumanHT12v4 gene chips. Next, downloading and reading expression profile data was conducted in R environment using GEOquery package^19^. Differential expression genes (DEGs) analysis was conveyed via Limma (linear models for microarray data) package^20,21^.

### Weighted gene correlation network analysis and the identification of modules

Considering the fact that the functions of most lncRNAs are unknown, the prediction of their functions principally depends on the examination of their co-expressed genes. Network analysis was conducted using the WGCNA package in R^22^ to evaluate the relative significance of lncRNAs and their module membership. Briefly, WGCNA was performed on the GSE106582 dataset obtained from 77 CRC and 117 mucosa tissues. To distinguish modules with different expression patterns, a soft threshold power was selected to create co-expression networks. Next, the pearson correlation coefficient was used to evaluate the weighted co-expression relationships in the adjacency matrix. Then, a topological overlap matrix (TOM) similarity function was applied to transform the matrix into a TOM, which was used to estimate the co-expression relationships between genes. The networks were established by merging genes with extremely similar co-expression patterns into modules. Consequently, the module with the key lncRNAs and their co-expressed genes was achieved. The reconstructed co-expression network was visualized using the Cytoscape software (version 3.7.0) and Cytohubba plugin (version 0.1)^23^.

### Functional annotation of the co-expressed genes in the module

Gene Ontology (GO) is a simple technique applied for annotating large numbers of genes to define attributes of gene products in three non-overlapping domains of molecular biology, including Molecular Function (MF), Biological Process (BP), and Cellular Component (CC)^24^. To identify genes and their corresponding functionalities, the Kyoto Encyclopedia of Genes and Genomes (KEGG) was employed to systematically analyze gene functions (www.kegg.jp/kegg/kegg1.html)^25^. To determine the potential functions of novel lncRNAs and their associated biological pathways, a functional enrichment analysis of their co-expressed genes was performed using the Fun Rich software (version 3.1.3).

### Statistical analysis

GSE106582 downloading and reading expression profile data was conducted in an R environment using the GEOquery package^18^. Differential expression genes (DEGs) were assessed between CRC and mucosal samples by empirical Bayesian method via t-test. DEGs analysis was conveyed via Limma (linear models for microarray data) package^19,20^. The cutoff criteria of the adjusted *p*-value (FDR) < 0.05 and | logFC | ≥ 0.5 were considered the threshold for significance to extract DEGs and DELs among 48.107 Probe sets. The top 2450 genes were evaluated with the critical value of the adjusted *P*-value < 0.05, and logFC ≥ |0.5|, were selected for further analysis. Subsequently, the DEGs were filtered by 5023 lncRNAs were retrieved from HGNC BioMart (https://biomart.genenames.org/) to detect differentially expressed lncRNAs (DELs).

## Results

### Identification of LncRNA candidates associated with colorectal cancer

**Figure 1:**
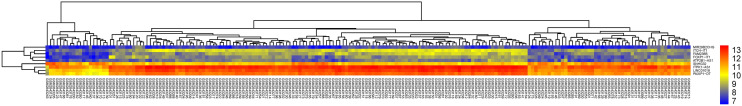
Identifying differently expressed genes between colorectal cancer and normal tissues. Heatmap of the difference between tumoral and normal samples of the GSE106582 dataset with R. Box-Scatter plot of the expression data of the lncRNAs in tumor tissues vs normal of the GSE106582 dataset for LINC01018.

GSE106582 gene expression profiles were selected in this study. This dataset contained 117 normal samples and 77 CRC samples. Following analysis of the dataset with the Limma package, the difference between CRC and normal tissues was presented in volcano plots (Fig. 1). Based on the criteria of adjusted *P*-value < 0.05 and logFC ≥ |0.5|, a total of 2449 DEGs were screened from GSE106582, including 1170 upregulated genes and 1279 downregulated genes. LncRNA expression data analysis of the GSE106582 dataset resulted in the identification of 32 DELs (Supplementary File 1), among which nine lncRNAs are detected as novel lncRNAs with no previous association with CRC development, including LINC01018, SNHG32, ITCH-IT1, ITPK1-AS1, FOXP1-IT1, FAM238B, PAXIP1-DT, ATP2B1-AS1, and MIR29B2CHG (Table 1). Of these, 8 lncRNAs were down-regulated (*p*-value < 0.05) and one lncRNA (SNHG32) was up-regulated (*p*-value < 0.05) in CRC tissues compared to normal tissues.

**Table 1:** Differentially expressed lncRNAs in colorectal cancer based on analyses of GSE106582 Dataset. Log2FC < 0: down-regulated, *From NCBI RefSeqGene.

Gene ID*	Official symbol	Official full name	logFC	Adj. *p*-value
50,854	SNHG32	Small nucleolar RNA host gene 32	0.737	2.76E−30
255,167	LINC01018	Long intergenic non-protein coding RNA 1018	− 0.738	9.34E−13
100,874,302	ITCH-IT1	ITCH intronic transcript 1	− 0.719	1.59E−14
319,085	ITPK1-AS1	ITPK1 antisense RNA 1	− 0.667	7.36E−14
100,506,815	FOXP1-IT1	FOXP1 intronic transcript 1	− 0.650	3.03E−15
731,789	FAM238B	Family with sequence similarity 238 member B	− 0.625	2.47E−10
202,781	PAXIP1-DT	PAXIP1 divergent transcript	− 0.600	1.89E−10
338,758	ATP2B1-AS1	ATP2B1 antisense RNA 1	− 0.565	6.76E−18
100,128,537	MIR29B2CHG	MIR29B2 and MIR29C host gene	− 0.536	1.81E−18

### Construction of weighted gene co-expression network analysis

**Figure 2:**
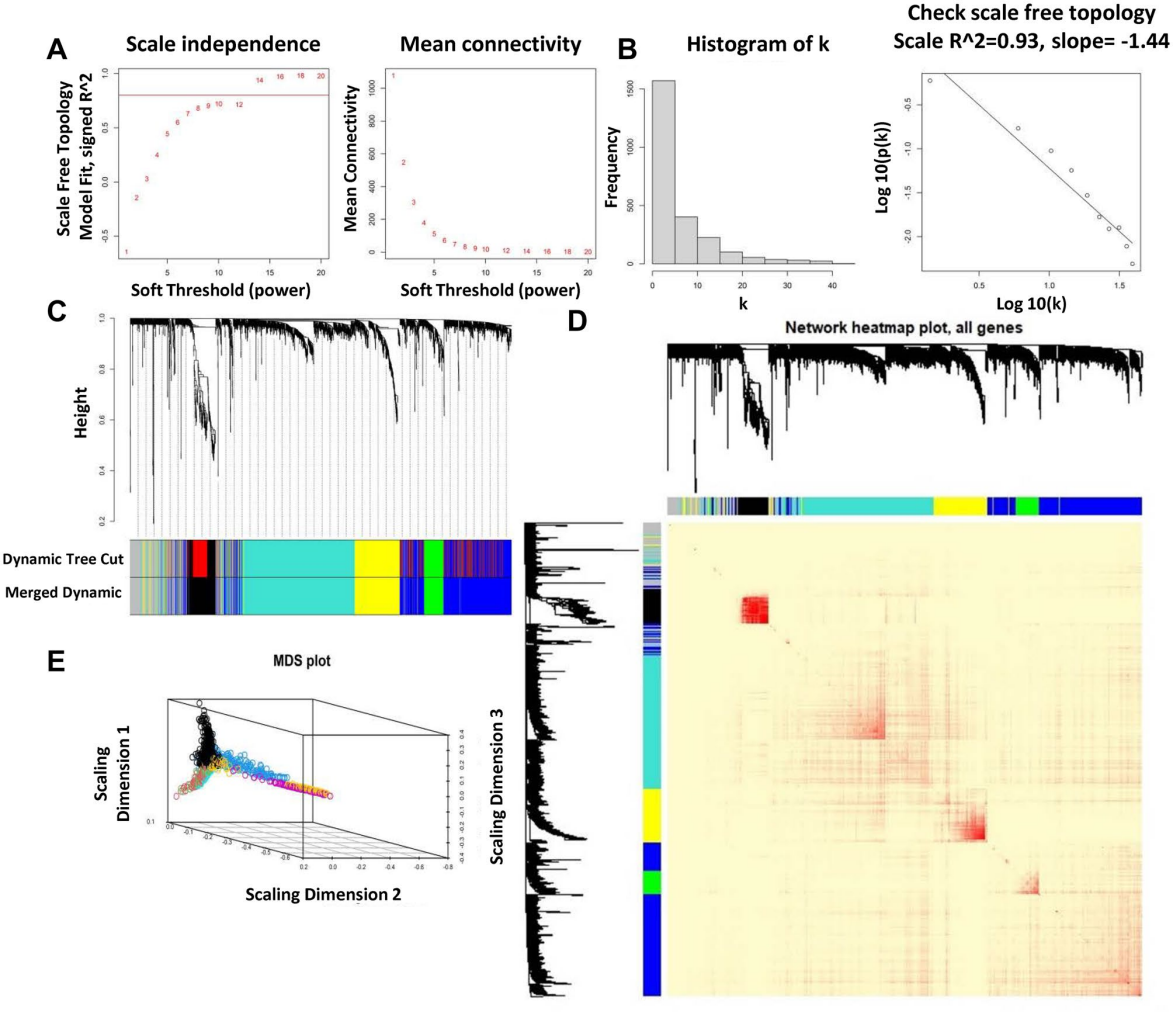
Network visualization plots. (**A**) Scale independence and mean connectivity analysis. The proper soft threshold power = 14 was selected. (**B**) The histogram of connectivity distribution and the scale-free topology panels. (**C**) Clustering dendrogram of genes, with dissimilarity based on the topological overlap. (**D**) Heatmap plot to represent the TOM among the genes in different modules. (**E**) Multidimensional scaling (MDS) plots to describe the entire gene expression network.

In this study, a co-expression network was constructed using GSE106582, the expression amounts of 2449 DEGs were analyzed for the co-expression network constructing the “WGCNA” package. Primarily, the outlier cases were displaced, and the hierarchical clustering analysis was accomplished with the “hclust” R function (Supplementary File 2). Meanwhile, the pickSoftThreshold function was used to determine scale independence and mean connectivity analysis of modules with several power values. Afterward, to guarantee a scale-free network, we picked β = 14 as the soft-thresholding power (Fig. 2A), to double-check the scale-free topology R2 with a linear regression plot (scale-free R2 = 0.93) (Fig. 2B). Therefore, β = 14 was selected to produce a hierarchical clustering tree with different colors signifying diverse modules. As demonstrated in Fig. 2C, six co-expressed gene modules were identified with gray modules representing non-co-expressed genes, and each module was marked by a color. The green module is the smaller module with 128 genes. At the same time, the blue module is the largest module with 780 genes. Additionally, the background color is grey and represents the 289 genes not attributed to any module (Table 2). Ultimately, we examined the interactive connections amongst the six modules, plotted the heatmap of the network, and showed the relative independence of each module in Fig. 2D and the multi-dimensional scaling (MDS) plot presented in Fig. 2E.

**Table 2:** Identified gene modules and their gene numbers.

Module colors	Gene numbers
Black	181
Blue	780
Green	128
Grey	289
Turquoise	753
Yellow	318

### Identification of novel lncRNA modules

We investigated the modules of novel lncRNAs to predict their functions through their co-expressed genes and the construction of a regulatory network between lncRNAs and protein-coding genes. We found LINC01018, ITCH-IT, ITPK1-AS1, FOXP1-IT1, FAM238B, and PAXIP1-AS1 in the black module, ATP2B1-AS1, and MIR29B2CHG in the turquoise module, and SNHG32 in the blue module. The list of genes for each module is detailed in Supplementary File 3.

### Gene co-expression modules correspond to CRC

**Figure 3:**
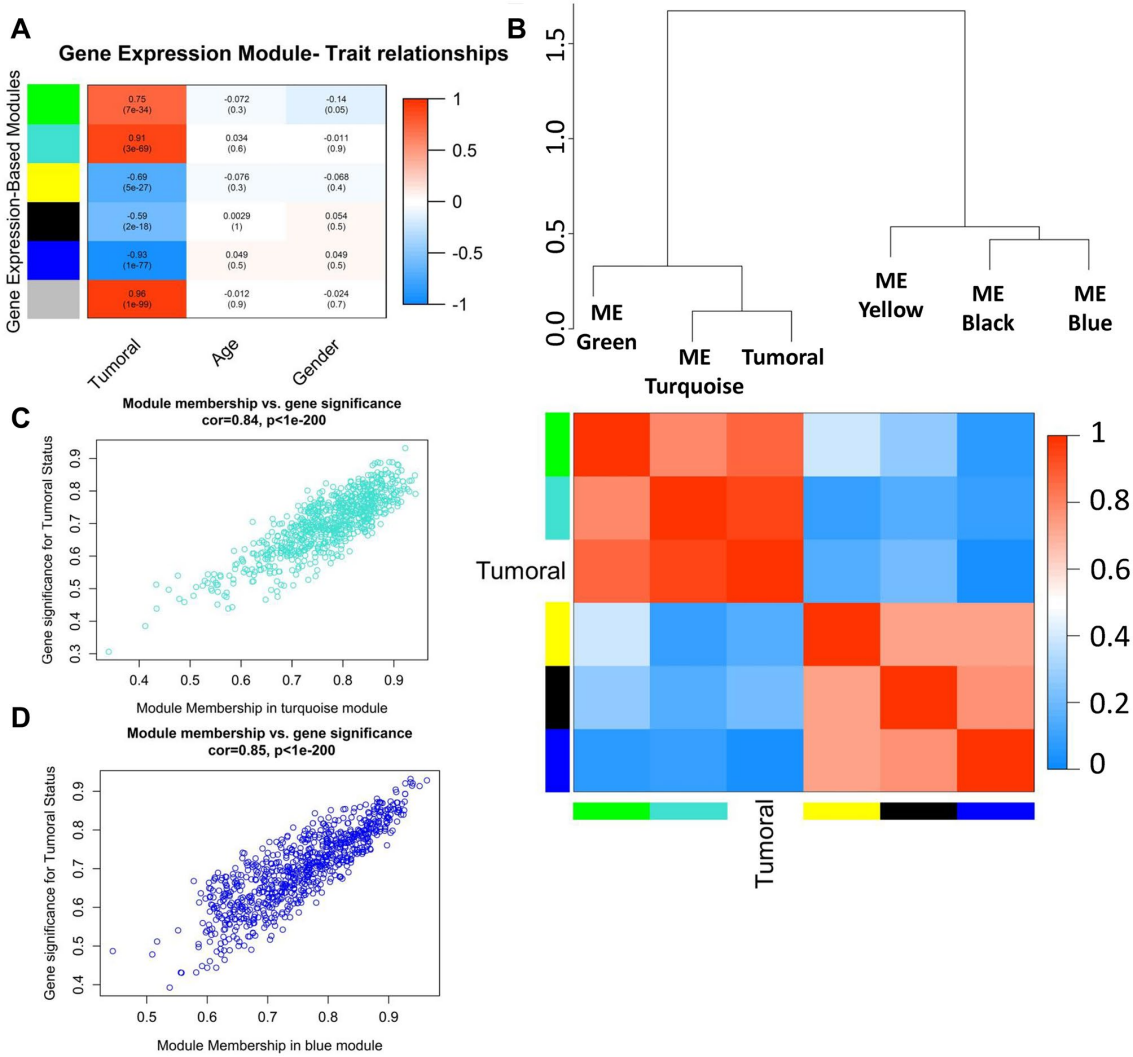
Gene co-expression modules correlated with colorectal cancer. (**A**) Module–trait relationships. Each cell includes the corresponding correlation and *P*-value. (**B**) The eigengene dendrogram and heatmap classify groups of correlated eigengenes. A scatter plot of the gene significance for Tumoral versus the module membership in the Turquoise (**C**) and Blue (**D**) modules.

In addition, we examined the associations of gene modules and cancer phenotype, which was based on the correlation between module eigengenes and clinical traits. The results revealed two of the total six gene modules were strongly correlated with tumoral status, including turquoise (R = 0.91, *P* = 3E−69), and grey (R = 0.96, *P* = 1E−99), while the grey module is non-co-expressed genes and not considered for further studies (Fig. 3A). In addition, the blue gene modules (R = 0.91, *P* = 3E−69) negatively correlate with tumoral status in a significant manner. However, other clinical traits, including age and gender, are not correlated with gene modules. Furthermore, the eigengene dendrogram and heatmap were designed to distinguish groups of correlated eigengenes associated with tumoral status. The results re-validated the correlations of the turquoise and blue gene modules with tumoral status (Fig. 3B). Finally, the plots of module membership in different gene modules vs. gene significance determined that the turquoise and blue gene modules have significant correlations with CRC and demonstrate these gene modules are associated with CRC (Fig. 3C, D).

### Construction of PPI network

**Figure 4:**
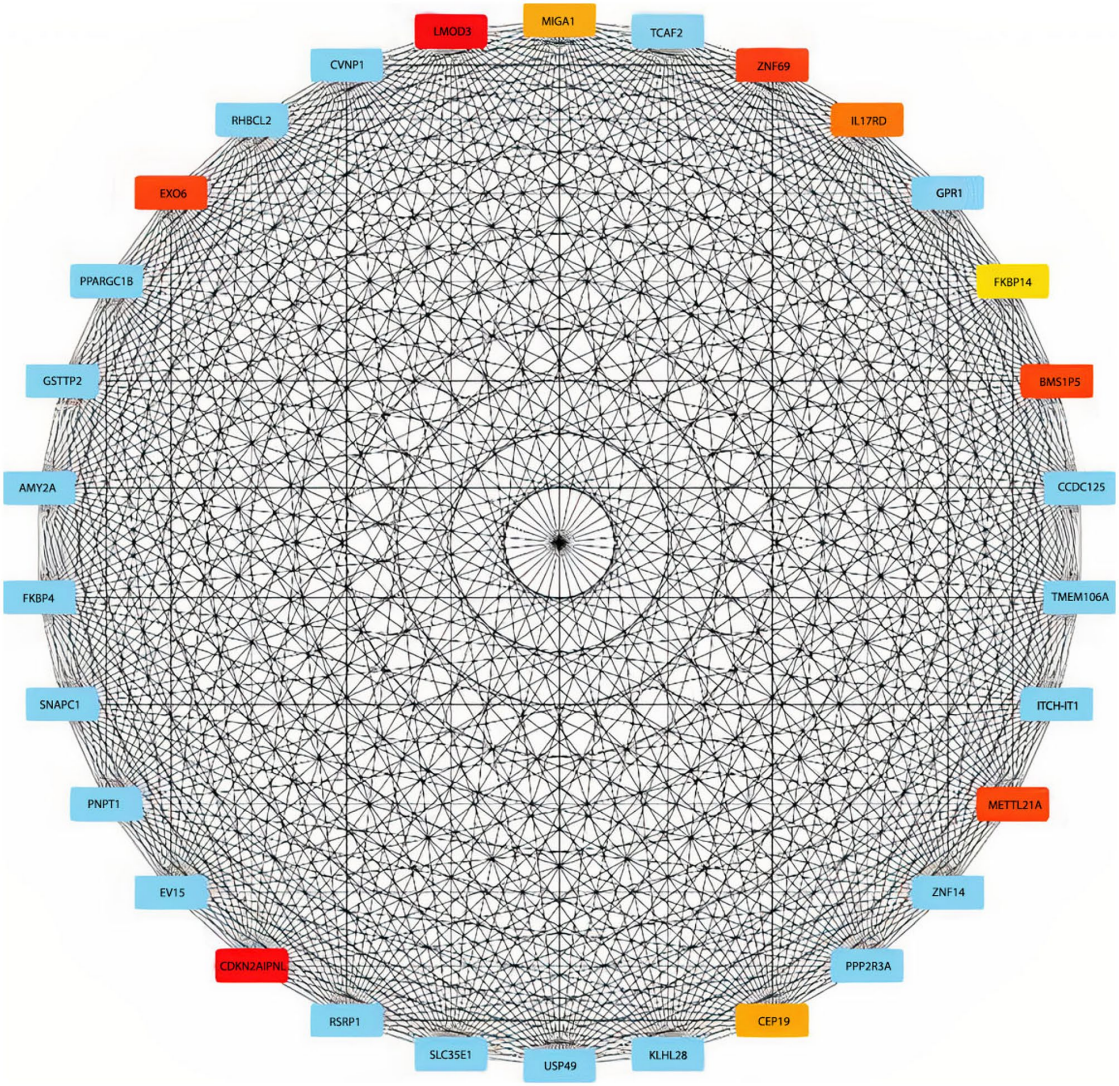
Black module network. The network of the top 30 genes or the most influential black module genes that are most closely related show the highest to lowest scores among these 30 genes in red, orange, yellow, and blue, respectively.

**Figure 5:**
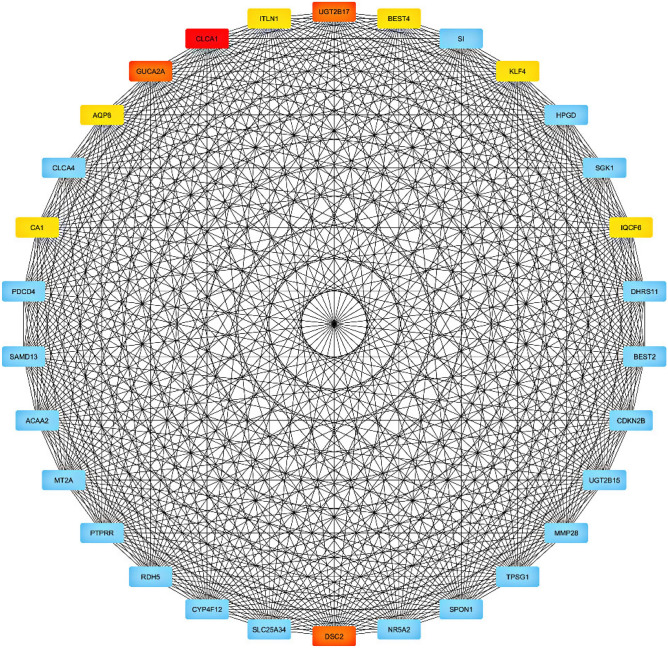
Blue module network. This module network analysis indicates that the highest score is related to the CLCA1gene.

**Figure 6:**
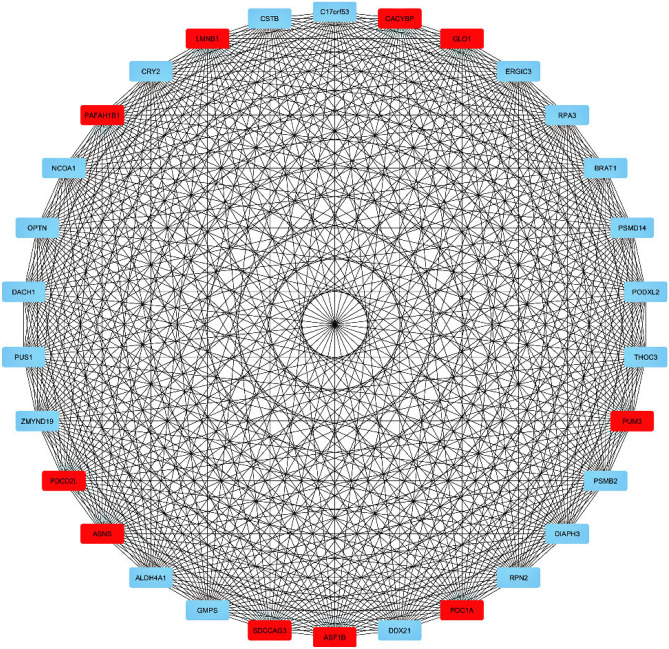
Turquoise module network. The top 10 genes in this module all have the same score and are equally involved in the respective pathways listed in Table 6.

CytoHubba plugin was used to construct interaction networks and identify hub genes in each module. Networks for black, blue, and turquoise modules are depicted in Figs. 4, 5 and 6, respectively. Moreover, the plugin has identified thirty hub genes based on their degrees, ranks, and scores that are summarized in Table 3.

**Table 3:** Identified hub genes for each module along with their ranks, and scores.

Black module			Blue module			Turquoise module		
Score	Name	Rank	Score	Name	Rank	Score	Name	Rank
11	LMOD3	1	4	CLCA1	1	1	PAFAH1B1	1
11	CDKN2AIPNL	1	2	GUCA2A	2	1	LMNB1	1
9	EXO5	3	2	UGT2B17	2	1	CACYBP	1
9	ZNF69	3	2	DSC2	2	1	GLO1	1
9	BMS1P5	3	1	CA1	5	1	PUM3	1
9	METTL21A	3	1	AQP8	5	1	POC1A	1
8	IL17RD	7	1	ITLN1	5	1	ASF1B	1
7	MIGA1	8	1	BEST4	5	1	SDCCAG3	1
7	CEP19	8	1	KLF4	5	1	ASNS	1
6	FKBP14	10	1	IQCF6	5	1	PDCD2L	1

### Functional enrichment analysis

The GO enrichment and KEGG pathway analyses were carried out to understand the biological characteristics of all modules. The involved cellular components, molecular functions, biological processes, and biological pathways for each module are summarized in Tables 4, 5 and 6.

**Table 4:** Black module functional enrichment results. www.kegg.jp/kegg/kegg1.html**.**

Gene ontology	*P*-value
**Cellular component**	
Nucleocytoplasmic shuttling complex	0.008314
Microtubule plus end	0.008314
DNA polymerase III complex	0.008314
Lysosome	0.013801
Hemidesmosome	0.01656
**Molecular function**	
Exonuclease activity	0.003126
Isomerase activity	0.006692
DNA binding	0.025009
Transcription regulator activity	0.026395
Amylase activity	0.042038
Protein domain specific binding	0.050233
**Biological process**	
Regulation of nucleobase, nucleoside, nucleotide and nucleic acid metabolism	0.036156
Mitochondrial transport	0.04204
Regulation of enzyme activity	0.04204
Regulation of metabolism	0.04204
DNA repair	0.088095
DNA replication	0.105682
**Biological pathway**	
Metabolism of RNA	0.002805
FGF signaling pathway	0.008288
Regulation of gene expression in beta cells	0.010298
Synthesis, Secretion, and Inactivation of Glucagon-like Peptide-1 (GLP-1)	0.010501
Incretin Synthesis, Secretion, and Inactivation	0.014174
p38 signaling mediated by MAPKAP kinases	0.014174

**Table 5:** Blue module functional enrichment results. www.kegg.jp/kegg/kegg1.html.

Gene ontology	*P*-value
**Cellular component**	
Exosomes	2.03E−09
Lysosome	4.38E−07
Peroxisome	7.42E−05
Extracellular	0.000231
Mitochondrial matrix	0.000511
Membrane fraction	0.001931
**Molecular function**	
Catalytic activity	2.2E−09
Transporter activity	0.001539
Hormone activity	0.003064
Oxidoreductase activity	0.006538
Antigen binding	0.006925
Intracellular ligand-gated ion channel activity	0.006942
**Biological process**	
Metabolism	1.56E−16
Energy pathways	4.29E−16
Transport	0.005432
Cell proliferation	0.020762
Ion transport	0.045855
Apoptosis	0.069383
**Biological pathway**	
Mesenchymal-to-epithelial transition	1.23E−15
Fatty acid, triacylglycerol, and ketone body metabolism	5.1E−08
Fatty acid beta-oxidation I	1.26E−06
Mitochondrial fatty acid beta-oxidation of Saturated fatty acids	1.25E−05
Mitochondrial fatty acid beta-oxidation	2.57E−05
Synthesis of Ketone Bodies	0.000427

**Table 6:** Turquoise module functional enrichment results. www.kegg.jp/kegg/kegg1.html.

Gene ontology	*P*-value
**Cellular component**	
Nucleolus	1.65E−38
Centrosome	4.24E−27
Nucleus	1.65E−25
Nucleoplasm	2.82E−22
Microtubule	2.86E−14
Mitochondrion	1.37E−13
**Molecular function**	
DNA-directed DNA polymerase activity	9.46E−05
Ribonuclease activity	0.000101
Structural constituent of ribosome	0.000383
Heat shock protein activity	0.000569
RNA binding	0.000796
DNA-directed RNA polymerase activity	0.000854
**Biological process**	
Regulation of nucleobase, nucleoside, nucleotide and nucleic acid metabolism	1.78E−09
Cell cycle	0.00149
Spindle assembly	0.001589
Energy pathways	0.002178
Metabolism	0.003503
Ribosome biogenesis and assembly	0.009037
**Biological pathway**	
Cell Cycle, Mitotic	2.97E−30
DNA Replication	1.53E−23
Mitotic M-M/G1 phases	1.28E−20
Mitotic G1-G1/S phases	6.1E−14
G2/M Checkpoints	1.72E−13
S Phase	1.75E−13

## Discussion

CRC is a global concern due to its high mortality and morbidity rates. Medical systems worldwide have been endeavored to reduce CRC rate using novel diagnostic and prognostic methods^26^. However, it is still one of the leading medical burdens over the globe^27^. High throughput technologies like microarray have been a valuable tool to compare the expression profile of normal and tumor cells. The omitted data has been precious to better understand expression alterations in cancers^28^. Using tools like WGCNA, we can study the interconnections between genes and obtain differentially expressed genes^29^.

In recent years, WGCNA has been used to comprehend lncRNAs role in cancers. Giulietti et al*.* identified eleven lncRNAs using this method as key regulators in pancreatic cancer, which could be used as novel diagnostic/prognostic markers^30^. Jiang et al*.* used the WGCNA method and found four lncRNAs associated with the carcinogenesis and progression of colon adenocarcinoma. In the current study, nine differentially expressed lncRNAs (LINC01018, ITCH-IT, ITPK1-AS1, FOXP1-IT1, FAM238B, PAXIP1-AS1, ATP2B1-AS1, MIR29B2CHG, and SNHG32) were identified using microarray data analysis (GSE106582). Afterward, WGCNA was performed on the lncRNAs and their target genes, which resulted in three significant modules. Further bioinformatics studies on hub genes of every module showed that they are involved in concrete pathways and biological processes.

The role of some of identified DELs in cancer pathogenesis has been previously reported in the literature. Miao et al*.* identified LINC01018 as a prognostic marker for gastric cancer^31^. It also has a tumor suppressor role in hepatocellular carcinoma that upregulates FOXO1 by sponging miR-182-5p^32^. In a recent study, Liting Wang et al. (2021) found that LINC01018 / hsa-miR-182-5p / ADH4 were strongly correlated. Moreover, the regulatory axis of ceRNA in the human body, by regulating the expression of key proteins in important signaling pathways can become a checkpoint inhibitor and regulate the incidence of liver cancer^33^.

In the study of Hu et al*.*, a combination of five lncRNAs, including ITPK1-AS1, was introduced as a useful prognostic marker for gastric cancer. A 2020 study also found that an ITPK1-AS1 anti-sensory ncRNA with 0.56-fold induction was the highest gene regulated by e-cigarettes compared to traditional cigarettes in active bronchial epithelial cells in smokers^34,35^. It is also a potential prognostic biomarker of colon adenocarcinoma^36^. Using bioinformatics tools, FOXP1-IT1 and other lncRNAs have been recognized as a useful prognostic marker for colon adenocarcinoma^37^. A study examining the expression pattern of different lncRNAs induced by TGF β1 predicts that FOXP1-IT1 is highly regulated by RAD21, possibly involved in oncogenic conversion and tumorigenesis in response to DNA repair and induction of genomic instability^38^. PAXIP1-AS1 is located in the glioma cell nucleus, and its overexpression increases migration, invasion, and angiogenesis of human umbilical vein endothelial cells in glioma^39^. Zhou et al., in a bioinformatics study, identified MIR29B2CHG as a useful prognostic marker for adrenocortical carcinoma^40^. A significant decrease of MIR29B2CHG was observed in the triple negative types of breast cancer. It is a host gene for producing of miR-29b2 and miR-29c, which plays a suppressive role in the progression of breast cancer^41^.

A co-expression network analysis using WGCNA between lncRNAs and their target genes resulted in black, blue, and turquoise modules. Enrichment and functional analysis using Cytoscape plugin Cytohubba indicated that the modules involved fundamental cellular pathways. Results showed black module was involved in the metabolism of RNA, FGF signaling pathway, and regulation of gene expression in beta cells; the blue module was mainly engaged in mesenchymal to epithelial transition, fatty acid, triglyceride, and fatty acid beta-oxidation, and the turquoise model played a critical role in mitotic cell cycle, DNA replication, and mitotic M-M G1 phase. Gene ontology study revealed modules participate in essential biological processes and molecular functions. For example, the genes of black and turquoise modules participate in regulating nucleic acid metabolisms. Moreover, they act in transcription regulation activity and DNA/RNA binding molecular functions. In The blue module, genes are involved in metabolism, energy pathway, and transportation biological processes. In addition, they function in catalytic, transporter, and hormone activities. The genes of black and blue modules are operating in the lysosomes and exosomes. Also, the turquoise module genes are located in the nucleus and mitochondrion.

Based on identified biological pathways, modules and hub genes might have an essential role in developing and malignancy of CRC. In the black module, Otte et al*.* detected the elevated expression level of several self-renewal and stemness-associated genes in cultures with active FGF2 signaling^42^. The p38 MAPKs are a family of serine/threonine kinases that mainly respond to external stresses^43^. They participate in significant cancer progression-related mechanisms, including cell metabolism, invasion, inflammation, and angiogenesis^44^. Glucagon-like peptide 1 (GLP-1) is secreted from intestinal L-cells and participates in insulin secretion and β-cell growth. It has been suggested that sustained activation of the GLP-1 receptor may indirectly result in colon cancer by hyperinsulinemia^45^. As other recognized pathways in the black module regulate gene expression in pancreatic beta cells and the synthesis/secretion of Incretin, there may be an association between insulin secretion, colon cancer, and genes in this module which needs to be further studied.

The genes in the blue module participate in the EMT process. The cells of the colon lose their epithelial trait and gain some mesenchymal characteristics that help them migrate to other parts of the body. This process is the main reason for liver metastasis that occurs in CRC patients^46^. In addition, it has been reported that lipid is required for cancer cells to proliferate. Due to this fact, pathways that participate in lipid synthesis would be proper targets to design therapeutic agents^47^. As summarized in Table 4, genes in the blue module participate in fatty acid, triacylglycerol, and ketone body metabolism. Fatty acid beta-oxidation is another identified pathway that cancer cells rely on for survival, stemness, metastasis, immune suppression, and drug resistance^48^. Considering all the above-mentioned discoveries, further study would elucidate the exact role of this module and lipid involvement in cancer.

The cytoHubba plugin has pointed out several hub genes for black (LMOD3, CDKN2AIPNL, EXO5, ZNF69, BMS1P5, METTL21A, IL17RD, MIGA1, CEP19, FKBP14), blue (CLCA1, GUCA2A, UGT2B17, DSC2, CA1, AQP8, ITLN1, BEST4, KLF4, IQCF6) and turquoise (PAFAH1B1, LMNB1, CACYBP, GLO1, PUM3, POC1A, ASF1B, SDCCAG3, ASNS, PDCD2L) modules. The number of identified hub genes has been previously reported to participate in colorectal cancer pathogenesis, downregulation of miR-193a-3p results in upregulation of IL17RD, which promotes colon cancer through inflammation^49^. Also, this protein promotes cancer by concealing cancer cells from immune surveillance^50^. As a pro-proliferation factor, Yang et al. indicated FKBP14 was upregulated in CRC tissues, which were associated with the poor prognosis of CRC patients^51^. CLCA1 has a tumor suppressor role by inhibiting the Wnt/beta-catenin signaling pathway and the EMT process. The study on human CRC samples indicates its expression has been significantly decreased^52^. Polymorphisms in the UGT2B17 gene have been associated with CRC risk in the Caucasian population^53^. Dsc2 is the only expressed member of the desmocollins family in the normal colorectal cell. A study on CRC cells shows Dsc2 is switched to Dsc1 and Dsc3 during cancer development^54^. Overexpression of AQP8 has significantly decreased CRC cell growth and metastasis^55^. Aleksandrova et al*.* indicated circulating ITLN1 concentration has been correlated with CRC risk^56^. KLF4 as a tumor suppressor inhibits colorectal cancer cell growth and is associated with poor overall survival^57^. A component of the ubiquitin pathway, CACYBP, is overexpressed in CRC patients and has increased cancer proliferation^58^.

## Conclusion

In the current study, with the help of bioinformatics tools, black, blue, and turquoise modules were regarded as the most critical modules in the progression and development of CRC. Moreover, thirty genes were recognized as hub genes that could be possible biomarkers for the diagnosis and prognosis of CRC. In addition, nine lncRNAs including, LINC01018, SNHG32, ITCH-IT1, ITPK1-AS1, FOXP1-IT1, FAM238B, PAXIP1-DT, ATP2B1-AS1, and MIR29B2CHG were identified with no previous association with CRC development which may serve important roles in the pathogenesis of CRC.

## Supplementary Information

Supplementary Information 1.

Supplementary Information 2.

Supplementary Information 3.

## Data Availability

All data generated or analysed during this study are included in this published article [and its supplementary information files].
