# Supplementary material for: Weighted correlation network analysis revealed novel long non-coding RNAs for colorectal cancer
Source: Sci Rep. 2022 Feb 22;12:2990. doi: 10.1038/s41598-022-06934-w (PMC8863977; doi:10.1038/s41598-022-06934-w)
Supplement: Supplementary file 2 — Supplementary Information 2. [file 41598_2022_6934_MOESM2_ESM.pdf]

s2:

Before removing outliers

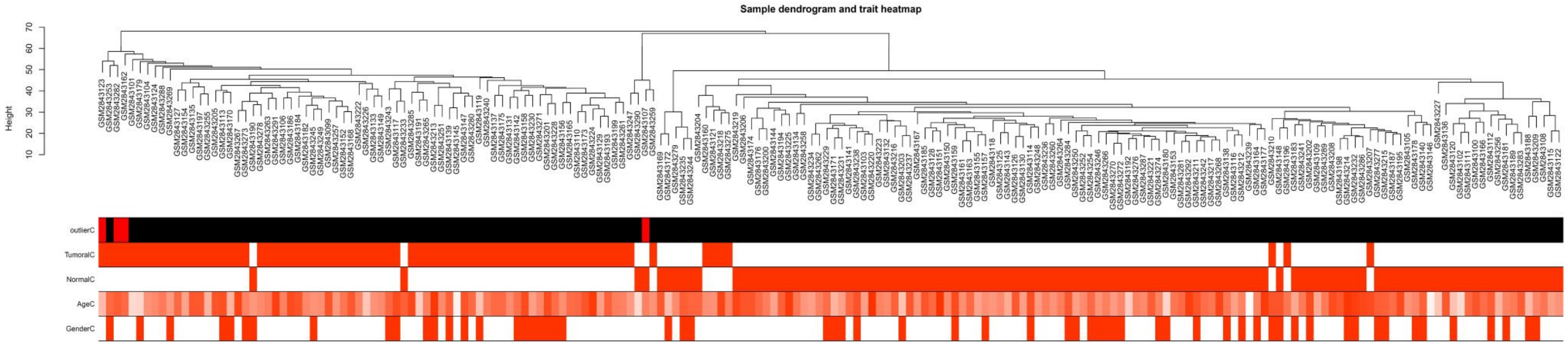

After removing outliers

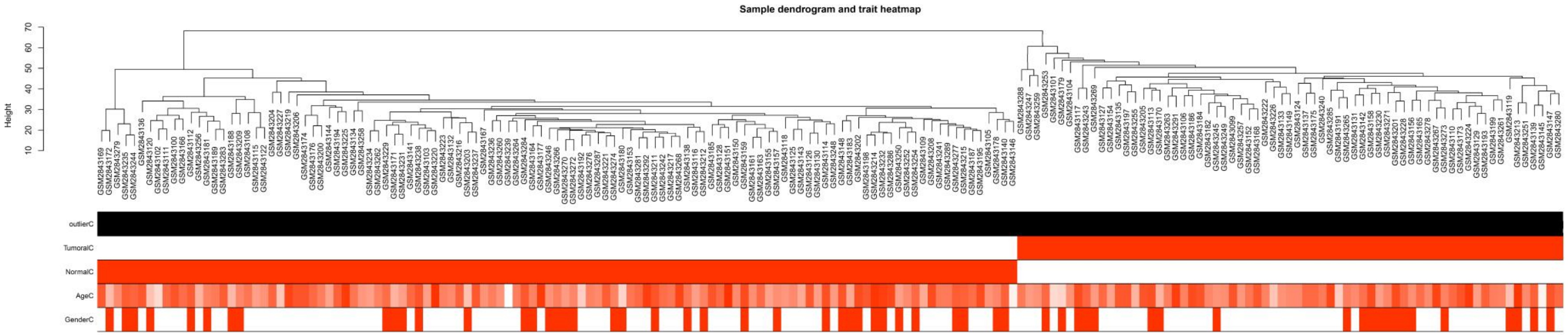

Supplementary file 2: Sample clustering and β decision.

Clustering dendrogram of samples based on their Euclidean distance. There were no outlier samples to remove.
